# Acid-Assisted Consolidation of Silver Alloys for Direct Fillings

**DOI:** 10.6028/jres.103.031

**Published:** 1998-10-01

**Authors:** Frederick C. Eichmiller, Kathleen M. Hoffman, Anthony A. Guiseppetti, Michael M. Wray, Rangall J. Avers

**Affiliations:** American Dental Association Health Foundation, Paffenbarger Research Center, National Institute of Standards and Technology, Gaithersburg, MD 20899-0001; Naval Dental School Bethesda, MD 20899-5602

**Keywords:** acid-assisted consolidation, cold-welding, consolidated silver

## Abstract

Silver-rich metal powders cold-welded by consolidation have been investigated as possible direct dental filling material. The surface of the silver powder must undergo an acid treatment to remove existing contaminants and prevent formation of new surface contaminants during consolidation. This study was designed to investigate the effect of the acid treatment on the strength of the consolidated alloy, its reactivity with the surrounding tooth structure, and its reactions with certain cavity liners.

This study investigated the effects of pH and concentration of fluoboric acid on the flexural strength of silver powder consolidated into rectangular beams in a partial 4 × 4 design. The study also assessed, by visible and scanning electron microscopy, what effect the acid-treated powders had on dentin that had been pre-coated with different cavity liners. Mean flexural strengths for beams consolidated using dental hand instruments were in the range (77.0 ± 9.28) MPa to (166.1 ± 17.6) MPa, where the quoted uncertainties are standard uncertainties (i.e., one standard deviation estimates). ANOVA indicated that fluoboric acid pH was highly significant (*p* < 0.0001) with lower pH values resulting in higher flexural strength. Concentration alone was not a significant factor for flexural strengths, but there was a significant interaction between concentration and pH (*p* < 0.0001). Microscopy revealed that the acid-treated silver powder demineralized approximately 2 μm of dentin when used with no liner. The use of copal or polyamide varnishes eliminated most of this demineralization, but the use of a dentin adhesive liner resulted in some dislodgment and breakdown of the adhesive film by the acid.

The results of this study indicate that this silver powder when treated with dilute fluoboric acid at a pH of approximately 1.0 can result in a filling material with strength equivalent to currently used conventional amalgam. The demineralization of tooth structure appears to be minimal and can be eliminated with the use of cavity liners.

## 1. Introduction

Amalgam has served effectively as a dental restorative material since its introduction into the United States by the Crawcour brothers in 1833 [1]. Recently, there has been much controversy over the use of amalgam due to documented hypersensitivities, accidental over-exposures and the potential environmental impact associated with its disposal [2,3,4]. In an attempt to reduce the release of potentially hazardous mercury, research has led to the development of indium- and gallium-based admixed alloys [5, 6]. Despite improved mechanical properties, these gallium and indium alloys have not been overwhelmingly successful [7, 8].

Since 1992, scientists from the American Dental Association Health Foundation and from the Electrochemical Processing Group of the National Institute of Standards and Technology have been collaborating on a new mercury-free dental filling alloy. This new alloy is a combination of powdered metals, consisting mostly of silver, that produce a filling material very similar in properties to those of cohesive gold used for directly placed restorations. The powdered metal is annealed, chemically treated to remove surface contaminants, and compressed into a solid mass. Welding of the individual oxide-free particles is accomplished by cold-welding the metal at room or body temperature, similar to the process used for cohesive gold. In addition to cold-welding, another unique characteristic of this system is the condensability of the powders under the liquid carrying medium. The powders are delivered as a slurry or mixture of metal particles within a dilute acid. The acid serves to both remove surface contaminants and protect the particles from reoxidizing during consolidation.

Effective cleaning of the powder surface may be related to many factors. A dilute solution of aqueous fluoboric acid (HBF_4_, diluted from an acqueous starting solution with a mass fraction of 48 %) with a volume fraction in the range of 2 % (pH = 0.97) to 10 %^1^ (pH < 1) has proven effective in facilitating cold welding, but the mechanism of surface cleaning and effect of acid pH or concentration are unknown. The purpose of the first part of this study was to determine which factors or combination of factors related to the activation acid would have the greatest effect on transverse rupture strength of the consolidated silver powder. The two primary factors studied were solution concentration of HBF_4_ and pH (H^+^ concentration) of the aqueous HBF_4_ solution.

Since the powder/acid slurry is consolidated directly into the tooth cavity, there exists the possibility of a reaction between the HBF_4_ and the tooth structure. Traditional cavity preparations are often lined with varnishes or adhesive polymers prior to placement of a filling material. The effectiveness of these liners in the presence of this powder/acid slurry, and possible reactions of the slurry with these liners are also unknown. Therefore, the purpose of the second part of this study was to determine if the dilute HBF_4_ acid would react significantly with the tooth dentin, and if the use of varnishes and liners would prevent any such reaction. Lastly, the use of lining materials in combination with the powder/acid slurry was tested to see if the presence of these liners would adversely affect the transverse rupture strength of the consolidated silver alloy.

## 2. Materials and Methods

The silver alloy powder used in this study was chemically precipitated using a reduction reaction in a solution of silver fluoroborate. After chemical precipitation, the silver powder was dried and sieved through a 200-mesh sieve to remove the large agglomerates. The sieved powder was then annealed for 2 h at 450 °C before activation in acid and consolidation.

### 2.1. Concentration and pH Effects

The precipitated silver alloy powder was activated by submersing a sample of the powder at mass 0.65 g in 350 mL of 10 % HBF_4_ (pH < 1) in a glass beaker and stirring it with a slowly rotating overhead stirring paddle for 1 min. The alloy was allowed to settle for approximately 4 min. The HBF_4_ was then decanted, leaving the settled powder as wet slurry. The slurry was then rinsed in 500 mL of a second solution of selected concentration and pH (see Table 1) and slowly stirred for approximately 5 s.

A total of 10 rinsing treatments were tested. The pH of rinse solutions 2 to 4 and 6 and 7 were adjusted by adding sodium hydroxide. Rinse solution number 8 was distilled water with 10 % HBF_4_ added to lower the pH to 2.57. The pH of 2.57 was chosen because residual 10 % HBF_4_ left from the activation in the powder slurry lowered the pH of the distilled water rinse to 2.57. Rinse solution number 9 was distilled water with 10 % HBF_4_ added to lower the pH to 2.57 then buffered with NaOH to bring the pH to 5. Rinse solution 10 repeated the distilled water rinse twice.

After the powder had settled again and the second rinse solution had been decanted, transverse rupture strength samples were made by transferring the remaining powder slurry to a steel mold with a rectangular cavity 14.01 mm in length by 3.01 mm in width by 20 mm in height. Consolidation of the silver powder was done by placing a single increment of the slurry into the base of the mold cavity and organizing the loose powder to form a flat, even layer. The powder was then pressed with a flat-ended plunger using a hydraulic press at a load sufficient to deliver an average stress of 150 MPa to the powder. This stress was chosen to match the density achieved by incrementally consolidating the same powder using hand dental instruments. The final thickness of the consolidated samples was approximately 1.5 mm. Five samples were consolidated for each activation/rinsing treatment. After consolidation, the samples were stored overnight in a desiccator and then dry polished in a longitudinal direction on 1200 grit silicon carbide paper until all surfaces were smooth and free of defects. Each sample was measured in all three dimensions and weighed. The polished samples were then tested on a three point bending fixture with a fulcrum spacing of 10 mm using an Instron 5500R universal testing machine (Instron Co., Canton, MA)^2^. Load was applied to the center of the sample at a constant crosshead speed of 0.5 mm/min until failure. Density and transverse rupture strength were calculated from the measured dimensions, weight and failure load of each individual sample. Transverse rupture strength (*TRS*), which is a stress (i.e., force divided by area), was calculated for each specimen from the equation *TRS* = 1.5 × 10^6^ *Fl*/*t*^2^*w*, where *F* is the load at failure, *l* is the distance between fulcrums (*l* = 10^−2^ m = 10 mm), *t* is the thickness of the specimen, and *w* is the width of the specimen. Data was analyzed by ANOVA and values of *TRS* compared using Tukey’s multiple comparison.

### 2.2 Evaluation of Lining Materials

Forty previously extracted, noncarious and nonrestored human molar teeth were mounted in Ultra Mount™ methyl methacrylate (Buehler Corp., Lake Bluff, IL) and stored in 0.015 mol/L sodium azide. Class I cavity preparations were then placed in the occlusal center of the teeth. The preparation was a “dogbone” retentive shape approximately 6 mm long and 2 mm wide with a 2 mm depth and roughly parallel walls. Each preparation was cut using a new #557 bur and air/water spray just prior to restoring. The teeth were divided into four equal groups. Each group received one of the following: no liner, copal varnish, polyamide varnish, or a polymer adhesive resin liner. The lining material was selected according to the order provided in a random number table to minimize operator bias.

The varnishes and adhesive were placed per manufacturer instruction as follows. Group 1: cavity preparation received no liner; Group 2: Copalite™ (H. S. Bosworth Co., Skokie, IL) copal resin was placed on the air dried preparation in two coats with 30 s air drying after each coat; Group 3: Barrier™ (Teledyne Getz, Elk Grove Village, IL) polyamide varnish was placed on the air dried preparation in one coat, left to dry for 15 s and then air dried; and Group 4: Mirage ABC™ (Chameleon Dental, Kansas City, KS) the air dried preparation was conditioned with 10 % phosphoric acid (H_3_PO_4_) for 15 s followed by rinsing with water and lightly air drying. Mirage primer A and primer B were then mixed and the adhesive resin was applied in five coats to the preparation, lightly air drying after each coat.

The experimental silver alloy was activated following the same method as in Sec. 2.1 with a rinse of 2 % HBF_4_ (pH = 0.97). The alloy was incrementally consolidated into five teeth of each lining group (total of 20 teeth) using a 1.15 mm diameter, serrated amalgam condenser under a load arising from approximately 2.5 kg to 3.5 kg. A load table was used to measure the load delivered to the condenser as it was stepped over the entire area of the sample in half overlapping uniform steps. Five or six increments were sequentially consolidated to completely fill the cavity. Excess HBF_4_ was removed by absorbing the acid off the surface of the silver alloy with cotton rolls after consolidating each increment. After consolidation was complete, the silver was burnished and excess silver was removed with rotary polishing burs.

The Tytin™ amalgam alloy was in capsule form and triturated per manufacturer instructions using a Vari-mix III™ amalgamator (Caulk/Dentsply, Milford, DE) for 9 s on medium speed. The amalgam was incrementally consolidated into the cavity preparation of 5 teeth of each lining group (total of 20 teeth) on the load table at a load arising from approximately 1.5 kg in the same manner as the experimental silver alloy. The consolidation process was repeated for approximately four total increments of the alloy or until the cavity preparation had been slightly overfilled. After consolidation was complete, the amalgam was burnished and excess was removed with carvers.

All tooth specimens were stored in distilled water for a minimum of 24 h at 37 °C after filling placement. Subsequently, each sample was sectioned with a diamond saw at 300 revolutions per minute using a high concentration diamond-wafering blade and distilled water lubrication. The samples were polished sequentially using (1200, 2400, and 4000) grit silicon carbide paper with distilled water lubrication on an optical polishing wheel. They were then polished with (6, 3, 1, and 1/4) μm Metadi™II diamond polishing compound (Buehler, Lake Bluff, IL) and DP-Lubricant Blue (Struers, Westlake, OH). The samples were rinsed with an ethanol stream and air-dried.

Each half of each tooth was examined and photographed using reflective light microscopy on a Olympus BH2™ microscope (Olympus Co., Lake Success, NY) with polarized light and a Nomarski differential interference contrast. A photomicrograph of a 0.01 mm calibration slide was taken for standardization of measurements from the reflective light microscope. After this examination, one half of each tooth was examined directly with a scanning electron microscope (SEM) and the other half indirectly. The halves for direct SEM observation were placed under vacuum for at least 1 week prior to examination in order to remove moisture. The half used for indirect observation was impressioned with Reprosil™ (Caulk/Dentsply, Milford, DE) hydrophilic vinyl polysiloxane impression material for the negative impression, and the positive replication made with Epon 828s (Shell Chem. Co., Houston, TX) low viscosity epoxy resin. The tooth halves and epoxy replicas were mounted on aluminum studs with carbon paint, gold sputtered using an Ultraspec 90 sputter coater (Energy Beam Sciences, Agawam, MA) and examined at 10 keV on a JSM 5300 SEM (JEOL, Peabody, MA). Photomicrographs were obtained along the pulpal floor of each restoration and measurements made to quantify any surface reaction zone present in the tooth dentin. The measurements were recorded and then averaged for each sample, along with a description in order to identify the location and characteristics of the reaction zone.

To investigate the possibility of contamination of the alloy powder by the liner or varnish, 20 experimental silver alloy samples were prepared for transverse rupture strength analysis. No liner, Copalite™ varnish, Barrier™ varnish, and Mirage™ adhesive (*n* = 5 for each method) were applied to the walls of a rectangular stainless steel split mold 14.01 mm × 3.01 mm. The silver powder alloy was prepared and consolidated into the mold incrementally using the same instruments and methods used in the extracted teeth. After the mold was filled and the sample was removed, stored for 24 h in a desiccator, and dry polished with 1200 grit silicone carbide paper until all surfaces were smooth and free of defects. Length, width, thickness and weight were recorded for each sample. The samples were tested for three point flexural strength by central loading in the same manner described in Sec 2.1.

## 3. Results

### 3.1 Concentration and pH Effects

The mean *TRS* and sample standard deviation for each treatment group is given in Table 2. An analysis of variance on all of the treatment groups showed pH having a significant effect at *p* < 0.0001 and the interaction between pH and concentration was significant at *p* < 0.0001. Concentration alone was not a significant factor. A Tukey’s multiple comparison at an alpha level of 0.05 resulted in groupings of similar treatments as indicated by the letters in Table 2.

### 3.2 Evaluation of Lining Materials

Scanning electron and light microscopy consistently revealed retention of the two varnishes under all conditions. There was a gap between the amalgam and tooth surface when no liner or no varnish was utilized. Generally, there was a narrow zone of demineralization in the samples restored with the new alloy and no liner. The adhesive liner had a visible reacted zone of dentin with both the new alloy and the amalgam which is consistant in appearence with an adhesive hybrid zone. The indirect replicate technique yielded no additional information. Table 3 is a summary of the observations and average thickness of the various lining materials in micrometers.

Transverse rupture strengths were recorded for the four groups of silver consolidated in the presence of the different liners and the mean values given in Table 4. There was no statistically significant difference between the treatments (analysis of variance at *p* ≤ 0.05).

## 4. Discussion

The results from Sec. 3.1 indicated that transverse rupture strength is not adversely affected until the rinse pH rises above 2.5. Concentration was not a significant factor, and it appears that using a rinse of 2 % HBF_4_ at the pH of 0.97 achieves adequate strength. The volumes of acid used for these activation experiments were very large and further experiments will need to be run to find the critical volumes, times, and stirring regimens necessary for activation. The surface cleaning of the powder, however, appears to provide for sufficient cold-welding to occur at this pH.

In Sec. 3.2, the varnishes appeared to remain intact on the dentin surface but there was evidence that the resin adhesive had been dislodged. Large inclusions of the resin were observed in some specimens that appeared as agglomerated polymer that had been removed from the cavity surface. The adhesive in the hybrid layer, however, was well retained. The resin adhesive liner caused the acid to rapidly produce a cloudy dispersion when the powder/acid slurry was condensed into the preparation. This may have been due to the hydrolytic degradation of the surface adhesive that was not entangled with the collagen of the hybrid layer.

Where no liner was used, the observed reaction zone appeared to be demineralized. The depth of this demineralization, approximately 2 μm, was less than that observed with the dentin adhesive and consistent with the demineralization experienced with etchants used with dentin adhesives [9]. The reason for this rather shallow level of demineralization was probably due to the short contact time that the acid had with the tooth surface during consolidation. The small volume of acid that could remain at the interface during consolidation and the high buffering capacity of the tooth surface would also limit the degree of demineralization that could occur. The adaptation of the alloy to this demineralized surface was very good and no open interfaces or contraction gaps were observed. The consolidated alloy did not undergo any shrinkage and did not exhibit the shrinkage gap observed in the amalgam restorations. No noticeable reaction was observed at any of the enamel/alloy interfaces.

According to Mertz-Fairhurst and Newcomer [10], an average amalgam/tooth interface gap with a dispersed phase amalgam was approximated at 13 μm with a sample standard deviation of 10 μm. Brannstrom [11] reported a 5 μm to 20 μm gap. These data appear consistent with the results obtained in this experiment. Eames and Hollenback [12] reported the average thickness of Copalite™ was 2 μm, and a thickness of 3 μm to 9 μm was reported by Dolven [13]. An average smear layer was reported to be 5 μm [11], again consistent with results presented here.

Results from scanning electron microscopy were difficult to interpret due to shrinkage artifacts introduced during desiccation of the tooth samples. Reflected light microscopy avoided the problem with drying artifacts and seemed to yield micrographs that gave a fair representation of tooth/silver interface.

Another concern with using liners was the possibility of the lining material reacting with the surface of the powder particles and preventing cold-welding. This was especially a concern in the case of the adhesive resin where it was observed that the acid became cloudy after contacting the cavity wall. The flexural strength measurements made on samples where the mold was lined with the varnishes and resin indicated that the liners had no effect on strength. Apparently, no reactions occurred to interfere with the cold-welding of the silver.

In an attempt to further investigate what was occurring with the various liners, a simple additional experiment was conducted. The experiment consisted of placing one coat of polyamide varnish, two coats of copal varnish and five coats of dentin adhesive onto separate glass slides. A drop of 2 % HBF_4_ was placed onto each of these slides. The adhesive was rapidly dislodged and disrupted from the slide while the two varnishes remained intact. This supplemental experiment helped confirm what was observed in the tooth samples.

## 5. Conclusions

From these experiments, it appears that activation of this silver powder can be adequately achieved using a regimen consisting of an initial immersion in 10 % HBF_4_ aqueous solution followed by a rinse in a HBF_4_ aqueous solution at a pH below approximately 2.5. A 2 % aqueous solution of HBF_4_ demineralizes the dentin cavity surface to a depth of approximately 2 μm when no cavity liner is used. Cavity varnishes and adhesive resin liners will prevent this demineralization. The adhesive on the surface of the cavity does not, however, remain adherent to the cavity wall in the presence of the powder/acid slurry. In addition, no shrinkage gap appeared between the cavity wall and the consolidated silver.

## Figures and Tables

**Table 1:** The rinse used in the second step for alloy consolidation

Rinse solution		pH	(volume fraction, %)
1.	HBF_4_ (no buffer)	< 1	10
2.	HBF_4_ (NaOH)	0.97	10
3.	HBF_4_ (NaOH)	2.57	10
4.	HBF_4_ (NaOH)	≈ 5.0	10
5.	HBF_4_ (no buffer)	0.97	2
6.	HBF_4_ (NaOH)	2.57	2
7.	HBF_4_ (NaOH)	≈ 5.0	2
8.	Single H_2_O (HBF_4_)	2.57	
9.	Single H_2_O (HBF_4_) and NaOH)	≈ 5.0	
10.	Double H_2_O	≈ 5.0	

**Table 2:** The mean and sample standard deviation of transverse rupture strength for each rinsing regimen (the letters indicate statistical grouping at *p* < 0.05 using Tukey’s multiple comparison)

Rinse solution			Mean *TRS* and sample standard deviation (MPa)	
1.	10 %	pH < 1	166.08 ± 17.58	(a)
2.	10 %	pH = 0.95	162.44 ± 14.59	(a)
3.	10 %	pH = 2.57	139.70 ± 19.45	(a,b)
4.	10 %	pH ≈ 5	77.04 ± 9.28	(d)
5.	2 %	pH = 0.95	151.76 ± 17.03	(a)
6.	2 %	pH = 2.57	115.28 ± 6.47	(b,c)
7.	2 %	pH ≈ 5	106.82 ± 13.86	(c)
8.	Single H_2_O rinse	pH = 2.57	113.23 ± 11.21	(b,c)
9.	Single H_2_O rinse	pH ≈ 5	118.11 ± 6.30	(b,c)
10.	Double H_2_O rinse	pH ≈ 5	94.64 ± 5.54	(c,d)

**Table 3:** Microscopy results

	Experimental alloy^a^	Amalgam^a^
No liner	2.2 (affected)	0.9 (smear layer) 5.4 (shrinkage gap)
Copal varnish	4.7 (varnish layer)	6.7 (varnish layer)
Polyamide varnish	5.1 (varnish layer)	7.2 (varnish layer)
Adhesive	6.9 (hybrid layer)	6.0 (hybrid layer)

a The numbers are layer thickness in μm; affected refers to the thickness of the demineralized zone of dentin.

**Table 4:** Mean and sample standard deviations of *TRS* values of silver consolidated in the presence of different liners

Varnish or adhesive	Mean *TRS* and sample standard deviation (MPa)
No liner	103.2 ± 8.3
Copal	103.0 ± 3.5
Polyamide	94.7 ± 12.0
Adhesive	91.7 ± 11.0

## Footnotes

1 All percent values hereafter are volume fractions.

2 Certain commercial equipment, instruments, or materials are identified in this paper to foster understanding. Such identification does not imply recommendation or endorsement by the National Institute of Standards and Technology or the American Dental Association Health Foundation, nor does it imply that the materials or equipment identified is necessarily the best available for the purpose.
